# Signaling by reactive molecules and antioxidants in legume nodules

**DOI:** 10.1111/nph.18434

**Published:** 2022-09-06

**Authors:** Samuel Minguillón, Manuel A. Matamoros, Deqiang Duanmu, Manuel Becana

**Affiliations:** ^1^ Departamento de BiologíaVegetal, Estación Experimental de Aula Dei Consejo Superior de Investigaciones Científicas Apartado 13034 50080 Zaragoza Spain; ^2^ State Key Laboratory of Agricultural Microbiology, College of Life Science and Technology Huazhong Agricultural University Wuhan 430070 China

**Keywords:** antioxidants, legume‐rhizobium symbiosis, nitrogen fixation, post‐translational modifications, reactive nitrogen species, reactive oxygen species, reactive sulfur species, redox signaling

## Abstract

Legume nodules are symbiotic structures formed as a result of the interaction with rhizobia. Nodules fix atmospheric nitrogen into ammonia that is assimilated by the plant and this process requires strict metabolic regulation and signaling. Reactive oxygen species (ROS) and reactive nitrogen species (RNS) are involved as signal molecules at all stages of symbiosis, from rhizobial infection to nodule senescence. Also, reactive sulfur species (RSS) are emerging as important signals for an efficient symbiosis. Homeostasis of reactive molecules is mainly accomplished by antioxidant enzymes and metabolites and is essential to allow redox signaling while preventing oxidative damage. Here, we examine the metabolic pathways of reactive molecules and antioxidants with an emphasis on their functions in signaling and protection of symbiosis. In addition to providing an update of recent findings while paying tribute to original studies, we identify several key questions. These include the need of new methodologies to detect and quantify ROS, RNS, and RSS, avoiding potential artifacts due to their short lifetimes and tissue manipulation; the regulation of redox‐active proteins by post‐translational modification; the production and exchange of reactive molecules in plastids, peroxisomes, nuclei, and bacteroids; and the unknown but expected crosstalk between ROS, RNS, and RSS in nodules.

**Table:** 

Contents		
	Summary	815
I.	Introduction	815
II.	Reactive oxygen species in nodule host cells	817
III.	Reactive oxygen species in bacteroids	822
IV.	Reactive nitrogen species in nodule host cells	823
V.	Reactive nitrogen species in bacteroids	826
VI.	Reactive sulfur species in nodule host cells	826
VII.	Reactive sulfur species in bacteroids	827
VIII.	Future perspectives	828
	Acknowledgements	828
	References	828

## I. Introduction

Plants produce an outstanding diversity of reactive molecules and antioxidants that are in continuous interplay in order to adapt and respond to environmental constraints and cues (Foyer & Noctor, 2013). Reactive oxygen species (ROS) include the superoxide anion radical (O_2_^−•^), hydrogen peroxide (H_2_O_2_), and hydroxyl radical (^•^OH), which derive from the reduction of O_2_ by one, two, and three electrons, respectively. Other important ROS in plants are singlet oxygen, formed from triplet chlorophyll at photosystem II under excess light, and alkoxyl and lipid peroxides, formed during membrane lipid peroxidation. Reactive nitrogen species (RNS) include nitric oxide (^•^NO), nitrogen dioxide (^•^NO_2_), *S*‐nitrosothiols (SNOs), and peroxynitrite (ONOO^−^). The term ‘nitric oxide’ (formally nitrogen monoxide) is commonly used to encompass not only the free radical but also the nitroxyl anion (NO^−^) and the nitrosonium cation (NO^+^), which are produced by the one‐electron reduction and oxidation of ^•^NO, respectively (Umbreen *et al*., 2018). The three molecular species are jointly represented as NO and that criterion will be followed here. More recently, a third set of reactive molecules, designated reactive sulfur species (RSS), has brought to the attention of plant biologists, following the interest raised in animals. This broad term includes hydrogen sulfide (H_2_S), sulfenic acid (RSOH), persulfides (RSSH), polysulfides (RS_*n*_H), disulfide‐*S*‐oxides (RSO_2_SR), and thiyl radicals (RS^•^), among other sulfur compounds (Gruhlke & Slusarenko, 2012). The best studied RSS is H_2_S, a lipophilic gas that operates as a signal molecule in animals and plants. It may occur also in the HS^−^ and S^2−^ forms but, because the concentration of S^2−^ is negligible at physiological pH, the effects of H_2_S may be ascribed to both H_2_S and HS^−^.

Most ROS, RNS, and RSS perform signaling roles in many physiological processes, including plant organogenesis and the perception and response to abiotic and biotic stress (Foyer & Noctor, 2013; Umbreen *et al*., 2018; Gotor *et al*., 2019; Smirnoff & Arnaud, 2019). These roles may be accomplished by conveying their bioactivity to proteins through post‐translational modifications (PTMs). Key redox‐related PTMs are carbonylation of lysine, proline, and other amino acid residues; nitration of tyrosine (Tyr); sulfoxidation of methionine; and various modifications of cysteine (Cys) residues, such as *S*‐nitrosylation, disulfide formation, *S*‐glutathionylation, sulfenylation, and persulfidation. However, to act as signals, the concentrations of reactive molecules need to be kept low and under tight control. Several antioxidant metabolites and proteins contribute to this purpose. Superoxide dismutases (SODs) catalyze the dismutation of O_2_^−•^ to H_2_O_2_ and are further classified into CuZnSOD, FeSOD, and MnSOD isoforms based on their catalytic metals (Becana *et al*., 1989; Rubio *et al*., 2004, 2007). Catalases are tetrameric hemoproteins that catalyze the decomposition of H_2_O_2_ to water and O_2_. Ascorbate and glutathione (GSH; γGlu‐Cys‐Gly) play multiple functions as redox buffers and also through the ascorbate‐GSH pathway to control H_2_O_2_ concentration in the chloroplasts and other cellular compartments (Foyer & Noctor, 2013). This pathway entails the concerted action of ascorbate peroxidase (APX), monodehydroascorbate reductase (MR), dehydroascorbate reductase (DR), and glutathione reductase (GR). Glutathione *S*‐transferases (GSTs) detoxify xenobiotics by their conjugation to GSH and scavenge peroxides using GSH as reductant (Frova, 2003; Dalton *et al*., 2009). Glutaredoxins (Grxs), and occasionally peroxiredoxins (Prxs) and glutathione peroxidases (Gpxs), are also reduced by GSH. All of them are encoded by large multigene families and may donate electrons to target proteins, thus transmitting redox signals (Rouhier, 2010; Dietz, 2011; Passaia & Margis‐Pinheiro, 2015).

Plant tissues also contain proteins involved in NO metabolism, the best studied of which are nitrate reductase (NR) and hemoglobins. Plant NR is a highly regulated enzyme that reduces nitrate (NO_3_^−^) to nitrite (NO_2_^−^) but also, under hypoxia, NO_2_^−^ to NO (Kaiser & Huber, 2001). Legumes contain two major types of hemoglobins: symbiotic or leghemoglobins (Lbs) and nonsymbiotic or phytoglobins (Glbs) (Becana *et al*., 2020; Berger *et al*., 2020b). Most if not all of them exhibit NO dioxygenase activity (NO + O_2_ → NO_3_^−^) and are therefore potentially involved in NO homeostasis. There are also enzymes that keep SNOs under control. *S*‐nitrosoglutathione reductase (GSNOR) catalyzes the NADH‐dependent reduction of *S*‐nitrosoglutathione (GSNO) to glutathione disulfide (GSSG). Because GSNO is a major NO reservoir in cells, GSNOR indirectly controls NO bioactivity in a plethora of processes. Thus, SNOs such as GSNO, formed by the reaction of NO with GSH, trigger *S*‐nitrosylation of proteins. For NO to act as a signal, the process should be reversible and, indeed, thioredoxin Trx*h5* was found to act as a selective protein‐SNO reductase (denitrosylase) in plant immunity (Kneeshaw *et al*., 2014). However, ONOO^−^ is generated by the reaction of NO with O_2_^−•^. Although ONOO^−^ does not react directly with Tyr, ONOO^−^‐derived radicals generate the tyrosyl radical (Tyr^•^), which in turn reacts with ^•^NO_2_ to yield 3‐nitrotyrosine (Radi, 2018). Based on the apparent irreversibility of Tyr nitration, ONOO^−^ has been considered only as a ‘highly oxidizing species’ and hence as a marker of oxidative damage. However, Tyr nitration may perform useful functions in plants by modulating protein activity or by inducing proteolysis, thus altering protein turnover and signaling cascades (Holzmeister *et al*., 2015).

The information about RSS in plants is much more scarce than in the case of ROS or RNS, but it is increasing rapidly as H_2_S is a signal molecule involved in many key processes from seed germination to organogenesis and fruit ripening (for a review see Gotor *et al*., 2019). In plants H_2_S production is linked to Cys metabolism and occurs at different cellular loci. The site of formation may be relevant for H_2_S bioactivity because cell membranes may slow down its transport resulting in local increases of concentration. The major routes of H_2_S generation are through sulfite reductase in the chloroplast, l‐desulfhydrase (l‐CDES) and d‐desulfhydrase (d‐CDES) in the cytosol, and β‐cyanoalanine synthase (CAS) in the mitochondria (Gotor *et al*., 2019).

**Fig. 1:**
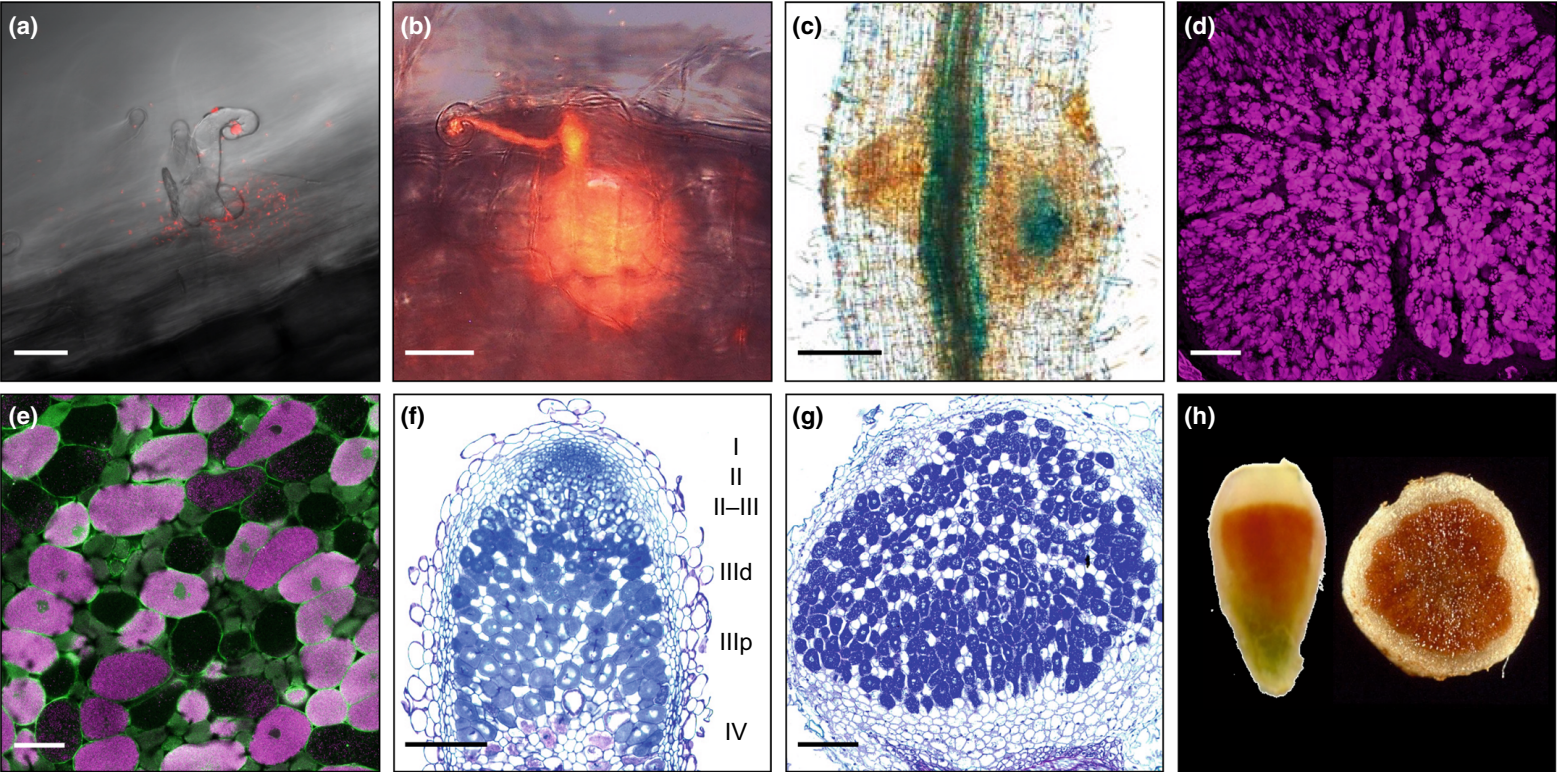
Legume nodule formation and development and distinctive structural features of indeterminate and determinate nodules. (a) Root hair of *Lotus japonicus* showing curling, entrapped bacteria, and incipient infection thread. The image was obtained 2 wk post inoculation (wpi) with *Mesorhizobium loti* strain MAFF303099 labeled with *Ds*Red. (b) Same symbiotic system as (a) showing a long infection thread and cortical cell division. (c) Nodule primordium of *L. japonicus* expressing GUS driven by the leghemoglobin *Lb2* promoter. Note blue staining in the infected cells. Image was taken at 5 d post inoculation (dpi). (d) Low‐magnification view of a determinate soybean nodule treated with nitrate showing bacteroids stained with SYTO 85 (nucleic acid stain; magenta). The image was taken at 4 wpi. (e) Similar view at high magnification showing infected cells replete of bacteroids and interstitial (uninfected) cells that appear ‘empty’. The image also shows that NO production (DAF‐2 DA; green) overlaps with bacteroids (SYTO 85; magenta). (f) Longitudinal section of an indeterminate nodule (*Medicago truncatula*) stained with toluidine blue and showing zone I (meristem), zone II (infection), interzone II–III, zone III (fixation; distal and proximal), and zone IV (senescence). Note some infection threads, especially in zone II and interzone II–III. (g) Section of a determinate nodule (*L. japonicus*) stained with toluidine blue. Note intense staining of infected cells. (h) Macroscopic sections of indeterminate (pea) and determinate (soybean) nodules. In the indeterminate nodule, the white, red, and green colors mark zones I–II, III, and IV, respectively. In the determinate nodule, the white and red colors mark the cortex and the infected zone. In both cases, the red color is due to the presence of millimolar concentrations of leghemoglobin. Bars: (a, b) 25 μm; (c, g) 100 μm; (d, f) 200 μm; (e) 50 μm. Credits and thanks: (a, b) Fukudome *et al*. (2016); (c, g) Wang *et al*. (2019); (d, e) Calvo‐Begueria *et al*. (2018); (f) Peter Mergaert (Institute des Sciences du Végétal, Gif‐sur‐Yvette, France); and (h) Larrainzar *et al*. (2020).

Reactive molecules and antioxidants are now known to be essential for symbiotic nitrogen fixation (SNF), a beneficial process for agriculture and the environment. Fig. 1 illustrates several steps during formation and development of legume nodules as well as their structural features. Legume roots are infected by soil bacteria, generically known as rhizobia, usually through a curl of the root hair forming an infection thread (IT) by which they reach the cortical cells and are released into the cytoplasm. The bacteria become engulfed in symbiosomes, organelle‐like structures surrounded by a membrane of plant origin, and are transformed into bacteroids that express nitrogenase and reduce (‘fix’) atmospheric nitrogen (N_2_) to ammonia that can be assimilated by the plant. As a result of infection, nodules are formed on the roots and in certain legume species also on the stems. There are two major types of nodules according to their growth pattern and tissue organization (Fig. 1). Indeterminate nodules, such as those of pea (*Pisum sativum*), vetch (*Vicia sativa*), white clover (*Trifolium repens*), and the model legume *Medicago truncatula*, have persistent meristems and show an elongate (quite often branched) form. They display a longitudinal gradient of age from the distal (apex) region to the proximal (base) region. The regions are designated as zones I (meristem), II (infection), III (fixation), and IV (senescence). Determinate nodules, such as those of soybean (*Glycine max*), common bean (*Phaseolus vulgaris*), cowpea (*Vigna unguiculata*), and the model legume *Lotus japonicus*, do not have persistent meristems and usually have a spherical form. They comprise a cortex at the periphery and a central zone containing infected cells and interstitial uninfected cells.

**Fig. 2:**
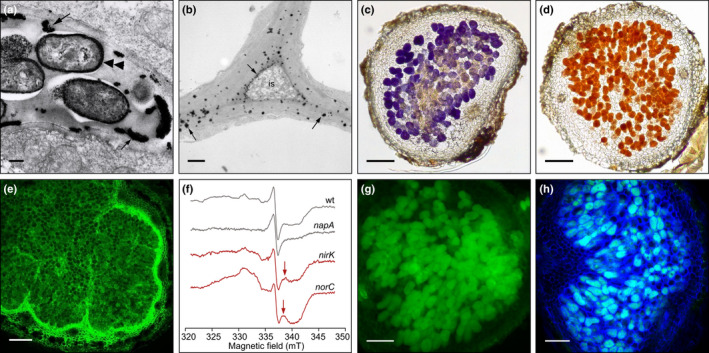
Selected methods used to localize production of reactive molecules in legume nodules. (a) Hydrogen peroxide (H_2_O_2_) (cerium chloride method). Transmission electron micrograph of a pea nodule (zone II) marking the accumulation of H_2_O_2_ (Ce^3+^ is oxidized by H_2_O_2_ to produce electron dense deposits of cerium perhydroxides) at patches in the periphery of the infection thread (arrows), as well as surrounding the bacteria within the infection thread (double arrowhead). (b) The same method as (a) was applied to show H_2_O_2_ accumulation in the intercellular space (is) in the inner cortex of a mature cowpea nodule. The matrix of the space and the cell walls that surround them contain abundant H_2_O_2_ (arrows). (c) Superoxide radicals (nitroblue tetrazolium) in a *Lotus japonicus* nodule deficient in the three Lbs (*lb123* mutant). Staining is observed in the infected zone. (d) Hydrogen peroxide (diaminobenzidine) in a nodule of the same mutant as (c). Staining is observed in the infected zone. (e) Detection of NO (4,5‐diaminofluorescein diacetate; DAF‐2 DA) in a soybean nodule produced by a *Bradyrhizobium diazoefficiens* strain lacking NorC. The green fluorescence is seen in the nodule parenchyma (cell layers of the cortex that surround the infected zone) and in the infected zone. (f) Detection by electron paramagnetic resonance spectroscopy of the nitrosyl‐leghemoglobin (Lb^2+^NO) complex. Arrows indicate the spectroscopic features marking the presence of Lb^2+^NO, and therefore NO production, in soybean nodules formed by the *B. diazoefficiens nirK* and *norC* mutants. Note that Lb^2+^NO was not detectable in nodules formed by the wild‐type strain or the *napA* mutant. (g, h) Detection of H_2_S and polysulfides with fluorescent probes in a nodule of *L. japonicus*. (g) HSip‐1 DA stain for H_2_S. (h) Merged image of calcofluor stain for cell walls (dark blue) and SSP4 stain for polysulfides (green); thus, in the merged image, polysulfides in the infected cells appear as light blue. Bars: (a, b) 150 nm; (c, d) 150 μm; (e) 200 μm; (g, h) 100 μm. Credits and thanks: (a, b) M. C. Rubio (CSIC, Zaragoza, Spain); (c) Rubio *et al*. (2004); (d) E. K. James (The James Hutton Institute, UK); (e, f) Calvo‐Begueria *et al*. (2018); (g, h) M. Fukudome and T. Uchiumi (Kagoshima University, Japan).

Here we will review both the known and the proposed roles of reactive molecules and antioxidants in SNF, from the early stages of nodule formation to nodule senescence. To facilitate reading, the information is organized separately for the three types of reactive molecules and for the two symbiotic partners. Although there is an exchange of signal molecules between the different compartments of nodule host cells and the bacteroids, as well as reactions between ROS, RNS, and RSS, we will tackle these issues, when pertinent, in the main text and figures. Another important consideration in signaling is the detection and quantification of reactive molecules due to their inherent instabilities and low concentrations. A summary of the methods most frequently used in studies of legume nodules is presented in Box 1 and some representative examples are shown in Fig. 2.

Box 1 **Methods for ROS, RNS, and RSS detection in legume nodules**

The methodology used to detect and localize reactive molecules in plant tissues needs to be defined in detail, requires strict controls, and in some cases is controversial. See main text for discussion on these issues. Here we summarize the methods that have been used in legume nodules.

1. The production of O_2_^−•^ is commonly detected by incubating nodule sections with nitroblue tetrazolium (NBT), which is reduced to a precipitate of blue formazan (Santos *et al*., 2001; Rubio *et al*., 2004;Andrio *et al*., 2013; Wang *et al*., 2019). Controls with O_2_^−•^ scavengers, such as 2,2,6,6‐tetramethyl‐1‐piperidinyloxy (TEMPO) or SOD mimics, are required to confirm specificity. The inhibitory effect of diphenylene iodonium (DPI) can be used to determine whether the source of O_2_^−•^ is NADPH oxidase (Andrio *et al*., 2013; Wang *et al*., 2019).
2. The production of H_2_O_2_ is also evidenced by cytochemical techniques using diaminobenzidine (light microscopy) or cerium chloride (electron microscopy) because these compounds are oxidized by H_2_O_2_ to produce brown or electron‐dense precipitates, respectively (Santos *et al*., 2001; Rubio *et al*., 2004; Andrio *et al*., 2013; Wang *et al*., 2019). Inhibitors of H_2_O_2_ production such as catalase, KCN, and ascorbate are usually included as controls. Alternatively, H_2_O_2_ sensors such as HyPer may be used in nodule sections with ratiometric fluorescent detection (oxidized form at 488 nm/reduced form at 405 nm). This method requires plant transformation (Andrio *et al*., 2013).
3. The production of NO in nodules may be (and should be) detected by complementary methods. The most widely used technique involves diaminofluorescein dyes in their diacetate membrane‐permeable forms. Once they are taken up by cells, esterases cleave the acetate groups and the resulting fluorescein reacts with dinitrogen trioxide (N_2_O_3_), an oxidation product of NO, to yield highly fluorescent triazoles. The dyes are highly sensitive for NO (*c*. 5 nM for 4,5‐diaminofluorescein (DAF‐2) and *c*. 3 nM for 4‐amino‐5‐methylamino‐2′,7′‐difluorofluorescein (DAF‐FM)) but differ in photolability and pH stability. Inhibition of the fluorescence signal by the NO scavenger, 2‐phenyl‐4,4,5,5‐tetramethylimidazoline‐1‐oxyl‐3‐oxide (cPTIO), is a minimal requirement to prove that NO is genuinely involved. Also, inhibitors like tungstate or arginine analogs are tested to obtain information on the source of NO (NR vs arginine‐dependent NO‐synthase activity), albeit interpretation of results must be extremely cautious. Alternatively, NO is evidenced in nodules by EPR spectroscopy, taking advantage of the paramagnetic properties of the nitrosyl complex between ferrous Lb and NO (Mathieu *et al*., 1998; Meakin *et al*., 2007; Calvo‐Begueria *et al*., 2018). This method is excellent for intact nodules but only allows detection of NO in the infected zone. EPR measurements require dedicated equipment and careful manipulation to avoid artifacts. Other methods are based on engineering NO biosensors in the bacterial partner by coupling an NO‐inducible promoter to a reporter gene. This approach can be used in combination with mutant strains that lack or overexpress Hmp, a bacterial NO‐detoxifying flavohemoglobin (Cam *et al*., 2012).
4. The SNO content is best measured by chemiluminiscence using an NO analyzer (NOA) before and after incubation with HgCl_2_ to release NO^+^ from SNOs (Chaki *et al*., 2011; Matamoros *et al*., 2020). The localization of SNOs is performed with the fluorescent reagent Alexa Fluor 488 Hg‐link phenylmercury (Molecular Probes, Eugene, OR, USA). Plant tissue sections are incubated in parallel in the absence of reductants and, as controls, in the presence of reductants (ascorbate and CuCl) that release the NO^+^ (Chaki *et al*., 2011). In both conditions, the thiol‐blocking agent *N*‐ethyl‐maleimide (NEM) is added before the probe to avoid interferences with SNOs.
5. Several RSS have been detected in nodules using fluorescent probes, sulfidefluor‐7‐acetoxymethyl ester (SF7‐AM) or HSip‐1 diacetate for H_2_S, and 3′,6′‐di(*O*‐thiosalicyl) fluorescein (sulfane sulfur probe 4; SSP4) for polysulfides, persulfides, and other sulfane sulfur compounds (Zou *et al*., 2019, 2020; Fukudome *et al*., 2020). SSP4 is applied in combination with cetyltrimethylammonium bromide to make it permeable. SF7‐AM is available from several suppliers, and HSip‐1 DA and SSP4 can be purchased from Dojindo (https://dojindo.com/product‐category/all‐products/sulfur‐biology/detection/).

## II. Reactive oxygen species in nodule host cells

Reactive oxygen species are involved at many stages of symbiosis, from rhizobial infection to nodule senescence. Table 1 is a compilation of the localizations of ROS in nodule tissues and the methods used to detect them. The formation of ROS was observed as early as the root perceives Nod factors. In *M. truncatula* the production of ROS and the expression of the *Rhizobium*‐induced peroxidase gene *rip1* were detectable after exposure of roots to compatible Nod factors (Ramu *et al*., 2002). In common bean, a transient (< 3 min) increase in ROS levels was seen at the tips of root hair cells within seconds after addition of Nod factors (Cárdenas *et al*., 2008). In this case, the response was shown to be specific for Nod factors because chitin oligomers failed to induce it and because the fungal elicitor chitosan caused a sustained increase in ROS.

**Table 1:** Localization of reactive molecules and methods used to detect and/or quantify them in legume nodules.

ROS/RNS/RSS	Localization	Legume	Method	References
O_2_^−•^	ITs, infected cells of young nodules	Alfalfa	NBT	Santos *et al*. (2001)
	Infected zone of Lb‐deficient nodules	*Lotus japonicus*	NBT	Wang *et al*. (2019)
H_2_O_2_	ITs, cell walls in infected zone	Alfalfa	Cerium chloride	Santos *et al*. (2001); Jamet *et al*. (2007)
	Intercellular spaces in infected zone	Soybean	Cerium chloride	Alesandrini *et al*. (2003)
	Matrix and walls of ITs, intercellular spaces in cortex, senescent zone	Alfalfa, pea	Cerium chloride	Rubio *et al*. (2004)
	Infection zone, inner cortex	*Medicago truncatula*	HyPer probe	Andrio *et al*. (2013)
	Infected zone	*L. japonicus*	DAB	Wang *et al*. (2019)
ROS	Infected zone of Lb‐deficient nodules	*L. japonicus*	DCFH‐DA	Wang *et al*. (2019)
NO	Young nodules	Soybean	EPR	Mathieu *et al*. (1998)
	Infected cells, bacteroids	*M. truncatula*	DAF‐2 DA	Baudouin *et al*. (2006)
	Nodules deficient in NirK or NorC, nodules under flooding stress	Soybean	EPR	Sánchez *et al*. (2010)
	ITs, nodule primordia, nodules deficient in bacterial Hmp	*M. truncatula*	DAF‐2 DA, biosensor strains	del Giudice *et al*. (2011); Cam *et al*. (2012)
	Nodules deficient in NorB	*M. truncatula*	DAF‐2 DA	Meilhoc *et al*. (2013)
	Nodules deficient in NirK or NorC	Soybean	EPR, DAF‐2 DA	Calvo‐Begueria *et al*. (2018)
SNOs	Mainly in infected zone and vascular bundles	Peanut	Alexa Fluor 488 Hg‐link phenylmercury	Maiti *et al*. (2012)
H_2_S	Infected zone of young and mature nodules	Soybean, *L. japonicus*	SF7‐AM, HSip‐1 DA	Fukudome *et al*. (2020); Zou *et al*. (2020)
RS_*n*_H	ITs, infected cells	*L. japonicus*	SSP4	Fukudome *et al*. (2020)

DAB, 3,3′‐diaminobenzidine; DAF‐2 DA, 4,5‐diaminofluorescein diacetate; DCFH‐DA, 2′,7′‐dichlorofluorescin diacetate; EPR, electron paramagnetic resonance; IT, infection thread; Lb, leghemoglobin; NBT, nitroblue tetrazolium; SF7‐AM, sulfidefluor‐7‐acetoxymethyl ester; SNOs, *S*‐nitrosothiols; SSP4, sulfane sulfur probe 4.

Several studies demonstrated the production of O_2_^−•^ and H_2_O_2_ in the ITs of indeterminate nodules. Specifically, H_2_O_2_ was found to accumulate surrounding the bacteria, in the thread walls, and in ‘patches’ in the thread matrices (Santos *et al*., 2001; Rubio *et al*., 2004; Jamet *et al*., 2007; Fig. 2a). Likewise, H_2_O_2_ was detected in the intercellular spaces and/or in their adjacent cell walls in the cortex of indeterminate (Rubio *et al*., 2004) and determinate (Fig. 2b) nodules. Based on inhibition and colocalization studies of CuZnSOD and H_2_O_2_, we proposed that H_2_O_2_ is formed, particularly in the ITs, by the sequential action of NADPH‐oxidase (also known as respiratory burst oxidase homolog; RBOH) and CuZnSOD (Rubio *et al*., 2004). In *M. truncatula* downregulation of *MtRbohA* decreased SNF (Marino *et al*., 2011), and, in common bean, downregulation of *PvRbohA* or *PvRbohB* impaired progression of ITs, suggesting that these enzymes are crucial for effective infection and release of rhizobia into nodule cells (Montiel *et al*., 2012; Arthikala *et al*., 2017). Another potential source of H_2_O_2_ in the ITs is diamine oxidase, which was proposed to be involved in crosslinking and subsequent hardening of the matrix glycoprotein in the lumen of ITs and in the intercellular spaces (Wisniewski *et al*., 2000). Notably, H_2_O_2_ was also detected surrounding the bacteroids in zone IV of indeterminate nodules and in the infected cells of determinate nodules, which is probably linked with the oxidative stress ensued during nodule senescence (Alesandrini *et al*., 2003; Rubio *et al*., 2004). This stress may involve Fe‐dependent Fenton reactions, as demonstrated *in vitro* with assays of deoxyribose degradation and lipid peroxidation and *in vivo* with an ^•^OH probe (Becana & Klucas, 1992).

Recent studies from our laboratories reported that *L. japonicus* nodules deficient in Lbs have an exacerbated production of O_2_^−•^ (Fig. 2c) and H_2_O_2_ (Fig. 2d) in the central, infected zone. Some of those nodules showed an heterogeneous distribution of O_2_^−•^, with higher staining at the periphery of the infected zone (Wang *et al*., 2019). Because of the lack of Lbs, this region may have a higher concentration of free O_2_ that results in an enhanced production of mitochondrial O_2_^−•^. However, ROS production was not inhibited by cyanide but by diphenylene iodonium (Wang *et al*., 2019), an inhibitor of NADPH oxidases and some other flavoproteins (Marino *et al*., 2011). These findings underline an important role of NADPH oxidases as a source of signaling O_2_^−•^ in nodule formation and metabolism, as well as the need to establish the spatio‐temporal profiles of expression of the multiple NADPH‐oxidase isoforms during nodule development. The study with Lb‐deficient nodules also suggests that Lbs might act as O_2_^−•^ scavengers (Wang *et al*., 2019). This appears to be at odds with the current perception that Lb is a major source of ROS in the cytosol of infected cells because the oxygenated form of Lb (Lb^2+^O_2_) can spontaneously autoxidize to ferric Lb (Lb^3+^) generating O_2_^−•^ (Puppo *et al*., 1981). To reconcile these two reactions, we propose that Lb acts in nodules, not only by transporting O_2_ to the bacteroids, but also as a ROS buffer, and that an equilibrium Lb^2+^O_2_ ↔ Lb^3+^ + O_2_^−•^ may exist *in vivo*. In addition, both Lb^2+^O_2_ and Lb^3+^ can trap H_2_O_2_, producing ferryl Lb and globin radicals that dissipate to more stable forms by formation of intramolecular and intermolecular heme‐protein crosslinks (Moreau *et al*., 1995). At high concentrations, H_2_O_2_ causes heme breakdown and release free Fe that can generate ^•^OH through Fenton reactions. This radical, in turn, reacts with polyunsaturated fatty acids to form lipid hydroperoxides and oxidizes amino acid residues of proteins. Indeed, carbonylation of a number of proteins, including Lb, has been recently detected in common bean nodules (Matamoros *et al*., 2018). The potential role of Lb as ROS scavenger and/or ROS generator *in vivo* deserves further investigation.

**Fig. 3:**
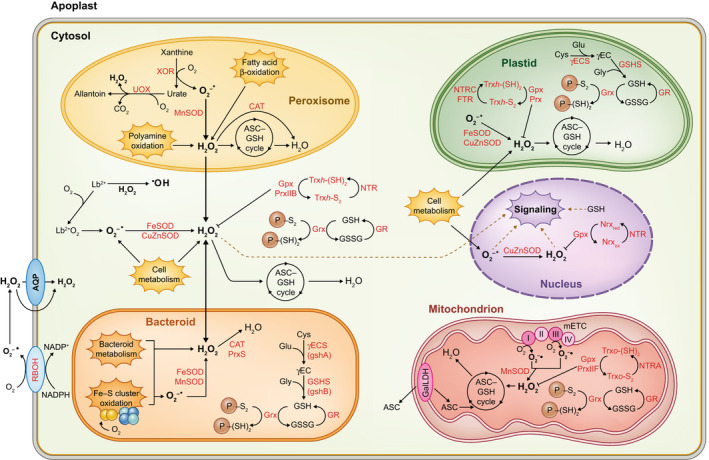
Reactive oxygen species (ROS) metabolism in legume nodules. The scheme is based on biochemical studies and on transcriptomic and proteomic data of *Lotus japonicus* (https://lotus.au.dk; Wang *et al*., 2019, 2022) and *Medicago truncatula* (https://medicago.toulouse.inrae.fr/MtExpress; Wienkoop *et al*., 2012). Among other ROS metabolic pathways in the bacteroids, the figure shows the generation of ROS through the irreversible oxidation by O_2_ of the Fe–S clusters of the nitrogenase enzyme complex. Dashed lines indicate potential signaling of ROS generated in the cytoplasm and conveyed to the nuclei. Blunt‐ended arrows indicate ROS scavenging. AQP, aquaporin; ASC, ascorbate; CAT, catalase; FTR, ferredoxin‐thioredoxin reductase; GalLDH, l‐galactono‐1,4‐lactone dehydrogenase; Gpx, glutathione peroxidase; GR, glutathione reductase; Grx, glutaredoxin; GSH/GSSG, reduced/oxidized glutathione; GSHS, glutathione synthetase; Lb^2+^/Lb^2+^O_2_, deoxyferrous/oxyferrous leghemoglobin; mETC, mitochondrial electron transport chain; Nrx, nucleoredoxin; NTR, NADPH‐thioredoxin reductase; Prx, peroxiredoxin; RBOH, respiratory burst homolog (NADPH‐oxidase); SOD, superoxide dismutase; Trx, thioredoxin; UOX, urate oxidase; XOR, xanthine oxidoreductase; γEC, γ‐glutamylcysteine; γECS, γ‐glutamylcysteine synthetase.

To keep ROS under control, preventing oxidative damage while allowing their signaling function, the nodule cytosol contains an impressive battery of antioxidants (Fig. 3). Cytosolic CuZnSOD (messenger RNA (mRNA) and protein) is preferentially found in the nodule apex whereas mitochondrial MnSOD (mRNA and protein) is abundant in the infected cells of indeterminate nodules (Rubio *et al*., 2004). Interestingly, the determinate nodules of cowpea and *L. japonicus* also contain a cytosolic FeSOD, which is increasingly expressed during nodule senescence. This increase is concomitant with a decline in Lb content and cytosolic CuZnSOD, which suggests a role of the Fe or heme released from Lb in the induction of FeSOD (Moran *et al*., 2003; Rubio *et al*., 2007). Ascorbate, GSH, and their associated enzyme activities are central for H_2_O_2_ homeostasis in nodules. The activities of APX and DR in the cytosol, as well as the nodule GSH content, were found to be positively correlated to SNF during nodule development (Dalton *et al*., 1986). The cytosol contains glutathione and homoglutathione synthetases, which catalyze the second step for the synthesis of thiol tripeptides (Moran *et al*., 2000; Frendo *et al*., 2001). There are also PrxIIB and Gpx, which metabolize H_2_O_2_ and lipid hydroperoxides, and are reduced back to the active forms by the concerted action of Trx*h* and NADPH thioredoxin reductase or, alternatively, by Grxs, GSH, and GR (Tovar‐Méndez *et al*., 2011; Alloing *et al*., 2018).

Nodule cells have high respiration rates to meet the energy demand of SNF. Moreover, respiratory consumption of O_2_ in the endodermis or nodule parenchyma may be an essential component of the O_2_‐diffusion barrier that regulates the entry of O_2_ into the infected zone to allow nitrogenase functioning (Minchin, 1997; Dalton *et al*., 1998). Like other plant organs, nodules generate copious amounts of ROS at the mitochondrial electron transport chain (mETC). The general view is that O_2_^−•^ is mainly generated by the univalent reduction of O_2_ at complexes I (NADH–ubiquinone oxidoreductase) and III (ubiquinol–cytochrome *c* oxidoreductase) by the ubisemiquinone intermediate (Rhoads *et al*., 2006). This O_2_^−•^ can dismutate to H_2_O_2_ either spontaneously or enzymatically. At complex III, O_2_^−•^ is formed on both sides of the inner mitochondrial membrane and easily reaches the cytosol through outer membrane porins. There is also evidence that ^•^OH is produced in the mitochondria (for review see Keunen *et al*., 2011). Mitochondrial ROS are likely candidates as signal molecules by which mitochondria regulate their metabolism and transmit information to the rest of the cell in a process known as retrograde signaling (de Souza *et al*., 2017). A fraction of these molecules might diffuse out of the mitochondria and be perceived by sensitive targets that relay the signal to other subcellular loci. Likewise, it has been hypothesized that oxidatively modified peptides and lipids produced in the mitochondria could reach the cytosol and act as selective secondary ROS messengers (Møller & Sweetlove, 2010; Farmer & Mueller, 2013).

To face oxidative stress and modulate ROS signaling, nodule mitochondria are endowed with diverse antioxidant systems (Fig. 3). The study of highly purified mitochondria from common bean nodules led us to propose the following model: O_2_^−•^ generated at the mETC is dismutated to O_2_ and H_2_O_2_ in the matrix by MnSOD; H_2_O_2_ is metabolized by APX in the inner membrane using ascorbate synthesized by l‐galactono‐1,4‐lactone dehydrogenase as electron donor; the resulting monodehydroascorbate and dehydroascorbate can be exported for its reduction in the cytosol or be reduced in the mitochondrial matrix by MR and DR (Iturbe‐Ormaetxe *et al*., 2001; Matamoros *et al*., 2006). There is also evidence to suggest the presence in nodule mitochondria of PrxIIF and Gpx1, which are involved in scavenging of H_2_O_2_ and lipid hydroperoxides (Ramos *et al*., 2009; Tovar‐Méndez *et al*., 2011).

Leaf chloroplasts are a major source of ROS as a result of photosynthetic electron transport and metabolism (Asada, 2006; Foyer & Noctor, 2013). They contain abundant antioxidant metabolites (ascorbate, GSH, carotenoids, and tocopherols) and enzymes (ascorbate‐GSH and GSH biosynthetic pathways, SOD, Prx, Trx, and Grx) (Rouhier, 2010; Dietz, 2011; Waszczak *et al*., 2018). Nonphotosynthetic plastids are expected to generate lower amounts of ROS than chloroplasts. Notwithstanding this, several antioxidants of chloroplasts are probably also operative in the nodule plastids because the enzyme activities have been assayed and/or because the corresponding genes have been found to be functional based on transcriptomic data (Fig. 3). It is also important to note that nodule proplastids and amyloplasts are crucial for SNF, being especially relevant for C and N metabolism in both ureide‐ and amide‐producing legumes (Robinson *et al*., 1994; Melo *et al*., 2003). Thus, the following antioxidant proteins were detected in nodule plastids: γ‐glutamylcysteine synthetase for GSH biosynthesis (Moran *et al*., 2000; Frendo *et al*., 2001), ferritin for Fe storage (Lucas *et al*., 1998), and an FeSOD isoform for O_2_^−•^ scavenging (Rubio *et al*., 2007). Leaf peroxisomes are also involved in crucial processes that generate ROS such as photorespiration, purine and polyamine metabolism, and fatty acid β‐oxidation (for recent reviews see Corpas *et al*., 2017; Sandalio *et al*., 2021). Typical peroxisomal enzymes producing high levels of O_2_^−•^ and H_2_O_2_ are, respectively, xanthine oxidoreductase and urate oxidase. To keep ROS under control and modulate their signaling roles, plant peroxisomes contain a variety of enzymes, which include catalase, the four enzymes of the ascorbate‐GSH pathway, MnSOD, and CuZnSOD (Corpas *et al*., 2017; Sandalio *et al*., 2021). However, very little is known about ROS signaling and antioxidants in nodule peroxisomes. Catalase is abundantly expressed in the infected zone of lupine (*Lupinus albus*) nodules (Lorenzo *et al*., 1990) and xanthine oxidoreductase and urate oxidase are located mainly to the interstitial cells of the central zone of soybean and cowpea nodules (Nguyen *et al*., 1985). The information on ROS production in the nuclei of nodule cells is very scant. H_2_O_2_ generated in other cellular compartments may reach the nucleus and act as a signal by interacting with nuclear proteins. To regulate this process, nodule cells contain Gpx in the nucleus (Matamoros *et al*., 2015). Gene expression analyses of the two model legumes also suggest the presence of nucleoredoxins. In Arabidopsis these proteins have dual nuclear/cytosolic location and the capability to reduce disulfides (Marchal *et al*., 2014). Immunolocalization studies revealed the presence of GSH in the nucleus and nucleolus of infected cells in pea nodules (Matamoros *et al*., 2013). This GSH may have important functions because its recruitment and sequestration in the nucleus during the G1‐ and S‐phases of the cell cycle have an impact on redox homeostasis and gene expression (Diaz Vivancos *et al*., 2010). Clearly, the role of plastids, peroxisomes, and nuclei of nodule host cells in ROS signaling and, in general, in SNF needs much more attention.

Several key nodule proteins are ROS targets as evidenced by the generation of oxidative PTMs, which suggests that redox regulation is important in symbiosis (Puppo *et al*., 2005; Oger *et al*., 2012; Matamoros *et al*., 2018). In *M. truncatula*, sulfenylated proteins were detected in roots at 2 d post‐inoculation (dpi) and in mature nodules at 4 wk post‐inoculation (wpi) (Oger *et al*., 2012). In the former case, the modified proteins are linked primarily to the redox state, whereas in the latter case they are rather associated to amino acid and carbohydrate metabolism. Another target protein for an oxidative PTM is malate dehydrogenase. This crucial enzyme for nitrogen assimilation in nodules was found to be carbonylated *in vivo* and its activity inhibited *in vitro* (Matamoros *et al*., 2018).

## III. Reactive oxygen species in bacteroids

Bacteroids, the N_2_‐fixing forms of rhizobia, are housed in symbiosomes within the infected cells. Indeed, crucial features of a functional symbiosis are the continuous exchange of metabolites and signal molecules between both partners through the symbiosomal membrane and the capacity of bacteroids to adapt to their peculiar microoxic environment. ROS, RNS, and probably RSS are key signal molecules involved in the host cell‐bacteroid communication and antioxidants are also critical to modulate their functions while preventing nitro‐oxidative damage (Box 2).

Box 2 **Redox signals and communication between bacteroids and host plant cells**

The onset of symbiosis requires a complex interplay between the host plant and the bacteria (Oldroyd *et al*., 2011). It is conceivable that a signal exchange continues in mature nodules, where symbiosomes behave as additional organelles, in order to integrate the metabolism of both partners and optimize SNF. But what is the nature of these signals?

1. The chemical properties of H_2_O_2_, NO, and H_2_S make them ideal candidates to transmit information between the host cells and the bacteroids. Antioxidant enzymes and metabolites may also participate in signaling directly and through regulation of reactive species.
2. Only molecules that are able to cross the symbiosomal membrane and possibly the bacteroid membranes may be effective signals. H_2_O_2_ cannot diffuse freely through membranes but aquaporins in the symbiosomal membrane could channel H_2_O_2_ in and out of symbiosomes (Clarke *et al*., 2014). NO is lipophilic and does not require a carrier to cross membranes (Domingos *et al*., 2015). H_2_S is also membrane permeable but at restricted diffusion rates (Filipovic *et al*., 2018). Specific sensors associated to the symbiosomal or bacteroidal membranes could perceive these molecules through PTMs of proteins and relay the information on the metabolic state of each symbiotic partner. Alternatively, peptides derived from the proteolysis of oxidized proteins, end‐products from the peroxidation of polyunsaturated fatty acids, or compounds derived from the modification of plant or bacterial metabolites by ROS, RNS, or RSS, might act as specific secondary messengers, as proposed for other plant organs (Møller & Sweetlove, 2010; Farmer & Mueller, 2013).
3. Bacteroids possess a high capacity for GSH synthesis and part of this GSH could be exported to the cytosol, where it may modulate the redox state and the activity of host proteins (for example, Grxs) for signaling purposes. Similarly, there is evidence that (h)GSH of plant origin is taken up by the bacteroids (Moran *et al*., 2000). However, GSH is unable to diffuse across lipid bilayers and transporters of GSH on the symbiosomal membrane have not been identified yet.
4. As occurs in pathogenic interactions, during infection rhizobial effector proteins are translocated to the host cell cytoplasm where they may help suppress the plant defense response (Walker *et al*., 2020). A similar mechanism might operate in mature nodules, where bacterial proteins might interfere with signal transduction pathways and thus regulate plant metabolism. Conversely, plant proteins and peptides may be targeted to the symbiosomes. This is the case of the nodule‐specific Cys‐rich peptides (NCRs) and Trx*s1*, which are present in nodules of legumes belonging to the inverted‐repeat lacking clade (IRLC). In *Medicago truncatula*, NCRs control rhizobia development and, in turn, Trx*s1* regulates the redox state of NCRs as well as bacteroid differentiation (Van de Velde *et al*., 2006; Ribeiro *et al*., 2017). Technological advances to isolate highly pure bacteroids and organelles and in‐depth proteomic analyses combined with *in vivo* specific labeling of proteins and metabolites will be necessary to demonstrate trafficking of signal molecules between the two partners inside the nodules.

Bacteroids contain a MnSOD (*sodA* gene) in the cytoplasm and, at least in the case of *Sinorhizobium meliloti*, also a CuZnSOD (*sodC* gene) in the periplasm. Actually, the product of *sodA* is a cambialistic SOD in *S. meliloti* (Santos *et al*., 1999) and *Rhizobium leguminosarum* (Asensio *et al*., 2011). These cambialistic enzymes can use either Mn or Fe as cofactors in their catalytic site, depending probably on metal bioavailability. It is unclear whether these rather unusual enzymes are also present in the bacteroids of determinate nodules or else if they contain a typical MnSOD or FeSOD. In any case, bacteroid SODs perform essential functions during nodulation. Thus, a *sodA*‐deficient mutant of *S. meliloti* nodulates poorly and displays abnormal infection, fails to differentiate into bacteroids, and enters senescence (Santos *et al*., 2000). Catalases have been examined in detail also in *S. meliloti*. This rhizobial species contains three enzymes with different biochemical properties and gene expression profiles: KatA and KatC are monofunctional catalases and KatB is a bifunctional catalase‐peroxidase (Jamet *et al*., 2003). KatA is the most abundant catalase in bacteroids and is inducible by H_2_O_2_, suggesting that bacteroids are naturally exposed to ROS and need to keep them under tight control. KatB is constitutively expressed in the bacteroids, and KatB and KatC are expressed in the bacteria within the ITs. All three single null mutants have no nodulation phenotype whereas the *katB katC* and *katA katC* double mutants show abnormal infection and poor nodulation (Jamet *et al*., 2003). Overexpression of KatB results in enlarged ITs, indicating that regulation of H_2_O_2_ is essential for optimal progression of ITs (Jamet *et al*., 2007). In contrast to *S. meliloti*, a single catalase, KatG, is responsible for catalase and peroxidase activities of *Rhizobium etli* bacteroids; however, this enzyme is not essential for nodulation or SNF in common bean (Vargas *et al*., 2003).

In bacteroids, like in plants, GSH is synthesized by two enzymes acting sequentially. Knockout mutants of several rhizobial species for γ‐glutamylcysteine synthetase (*gshA*) and glutathione synthetase (*gshB*) genes display impaired root colonization, delayed nodulation, and early nodule senescence (see further details in Mandon *et al*., 2021). Also, mutants of *S. meliloti* deficient in GR (*gor*) exhibited delayed nodulation and reduced SNF. The mutants showed induction of GSH biosynthetic enzymes as well as of KatA and KatB, reflecting alterations in redox homeostasis (Tang *et al*., 2017). All these findings underline an essential role of GSH in SNF. Besides the importance of GSH and the GSH/GSSG redox couple by themselves, GSH is also a substrate of several enzymes. Bacteroids contain two types of Grxs. In *S. meliloti* Grx1 (‘dithiol or class 1’ Grxs) plays a role in protein deglutathionylation, whereas Grx2 (‘monothiol or class 2’ Grxs) is involved in Fe metabolism (Mandon *et al*., 2021). These authors showed that the *grx1* mutant produces abortive nodules and undifferentiated bacteroids, whereas the *grx2* mutant has reduced SNF capacity but fully differentiated bacteroids.

In rhizobia other enzymes may catalyze the reduction of H_2_O_2_ and/or organic peroxides. The information is scattered but a few examples can be mentioned, such as PrxS (atypical 2‐Cys Prx) in *R. etli* and AhpCD (alkylhydroperoxide reductase) and Ohr (organic peroxide resistance) in *Azorhizobium caulinodans*. Mutants defective in PrxS remain unaffected in their symbiotic capacity but the *prxS katG* double mutant shows > 40% reduction in SNF, suggesting functional redundancy (Dombrecht *et al*., 2005). However, a role for AhpC (a Prx enzyme) in H_2_O_2_ detoxification *in vitro* and in protecting nitrogenase of stem nodules (but not of root nodules) has been reported (Jiang *et al*., 2019). Also, *ohr* mutants have hypersensitivity to organic hydroperoxides, fewer stem nodules, and decreased nitrogenase activity, underscoring a protective role of the protein in SNF (Si *et al*., 2020). Because the null mutants of all these genes show a nodulation phenotype and lower SNF, further investigation on the mechanisms of action of the protein products during nodule formation and senescence is warranted.

## IV. Reactive nitrogen species in nodule host cells

The discovery that NO is a key molecule in the plant–pathogen interaction (Delledonne *et al*., 1998; Durner *et al*., 1998) boosted studies on its role in the legume–rhizobium symbiosis because the two types of interactions share some signaling components (Hayashi & Parniske, 2014). For obvious reasons, NO is the RNS most extensively investigated in symbiosis and has been reviewed in detail (Meilhoc *et al*., 2011; Hichri *et al*., 2015; Berger *et al*., 2021). Therefore, we will provide here only the most relevant information about the established data and the controversial issues of NO in symbiosis, and will end this section by giving some brief information on other RNS.

The methods used for the detection and localization of NO in legume nodules are indicated in Box 1 and Table 1. All of them have pros and cons and it is most important to confirm NO production by two completely different methods; for example, fluorescent probes (Fig. 2e) and electron paramagnetic resonance (EPR) spectroscopy (Fig. 2f). The use of cell‐permeable probes requires nodule sectioning, incubation of sections with the probes at room temperature, and strict controls for NO specificity. Actually, the reactive molecule is N_2_O_3_, which is formed by the reaction of NO with O_2_. The advantage of fluorescent probes is that they permit to localize NO throughout the nodule, but sectioning drastically alters the microoxic conditions prevailing inside the nodules. The EPR method detects the Lb nitrosyl complex (Lb^2+^NO) in intact nodules (Mathieu *et al*., 1998; Meakin *et al*., 2007; Calvo‐Begueria *et al*., 2018). It is critical to harvest the nodules in liquid nitrogen and immediately pack them into the EPR quartz cuvette, so that artifacts are avoided. This method preserves nodule integrity (especially *in vivo* O_2_ concentrations) and is specific for NO, although it only allows localization of NO in the infected zone. Bearing these caveats in mind, it has been shown that NO is formed at virtually all stages of the symbiosis, from root hair infection to nodule senescence (Table 1). NO is transiently produced in the roots of *L. japonicus* and *M. truncatula* after 4 and 10 h, respectively, of inoculation with their symbiotic partners *Mesorhizobium loti* (Nagata *et al*., 2008) and *S. meliloti* (Berger *et al*., 2020a,b). This ‘NO burst’ is considered to be an initial plant defense response, which is suppressed once the bacteria have been perceived by the plant as symbionts rather than pathogens. In fact, when the roots are challenged with pathogenic bacteria, the NO burst is extended for at least 24 h, triggering a massive and sustained defense response. How the NO burst is induced and, shortly after, suppressed during infection with symbiotic bacteria is still not fully understood. At least one component of the induction response is the rhizobial lipopolysaccharide (LPS) because the application of LPS or its derived fractions, purified from *M. loti*, triggers an increase of NO and of the expression of a class 1 Glb in the roots of *L. japonicus* (Murakami *et al*., 2011). Class 1 Glbs, such as those of *L. japonicus* (LjGlb1‐1) and *M. truncatula* (MtGlb1‐1), are involved in NO scavenging to suppress the defense response of the plant (Nagata *et al*., 2008; Fukudome *et al*., 2016; Berger *et al*., 2020b).

After successful infection, NO can be detected in root hairs, ITs, nodule primordia, and zones III and IV (del Giudice *et al*., 2011; Hichri *et al*., 2015). In mature nodules, NO is detected by EPR under hypoxic conditions induced by waterlogging (Sánchez *et al*., 2010; Calvo‐Begueria *et al*., 2018). The use of transgenic legumes with altered expression of LjGlb1‐1 or MtGlb1‐1 has led to the conclusion that these proteins are central in the regulation of symbiosis and, hence, of SNF (Fukudome *et al*., 2016; Berger *et al*., 2020b). In *L. japonicus*, transient or stable overexpresion of *LjGlb1‐1* decreases the NO level, enhances the nodule number and SNF, and delays nodule senescence (Shimoda *et al*., 2009; Fukudome *et al*., 2019). Conversely, the *LjGlb1‐1* knockout lines showed a greater transient accumulation of NO in the inoculated roots than the wild‐type plants, with a reduction in the number of long ITs and nodules (Fukudome *et al*., 2016). In *M. truncatula*, high NO promotes defense responses and nodule organogenesis, whereas low NO promotes the infection process and nodule development (Berger *et al*., 2020b). These authors concluded that a tightly regulated concentration of NO is required at each stage of symbiosis. In other words, too much NO or too little NO are both detrimental to symbiosis.

But what are the sources of NO and the mechanisms of NO homeostasis in nodules? Traditionally, the pathways for NO production in plant cells are classified as ‘oxidative’ or ‘reductive’ (for a recent review see Astier *et al*., 2018). The major oxidative pathway in plants may involve the oxidation of the guanidino group of arginine to NO forming citrulline, as occurs for the animal NO synthase (NOS) isoforms. However, a genuine NOS activity has been found only in certain algae and not in land plants (Astier *et al*., 2018). The activity in extracts from higher plants is generically known as ‘NOS‐like activity’ as it is dependent on arginine, NADPH, and similar cofactors to animal NOS. It has been detected by chemiluminescence or EPR in peroxisomes and chloroplasts, but the identities of the responsible enzymes remain elusive. An earlier report concluded that the roots and nodules of white lupine have NOS‐like activity (Cueto *et al*., 1996). This conclusion should nevertheless be reassessed because the techniques used to demonstrate NO production were indirect and nonspecific. Clearly, a direct method to detect NO is required to unequivocally demonstrate the presence of arginine‐dependent, NOS‐like activity in legume nodules.

**Fig. 4:**
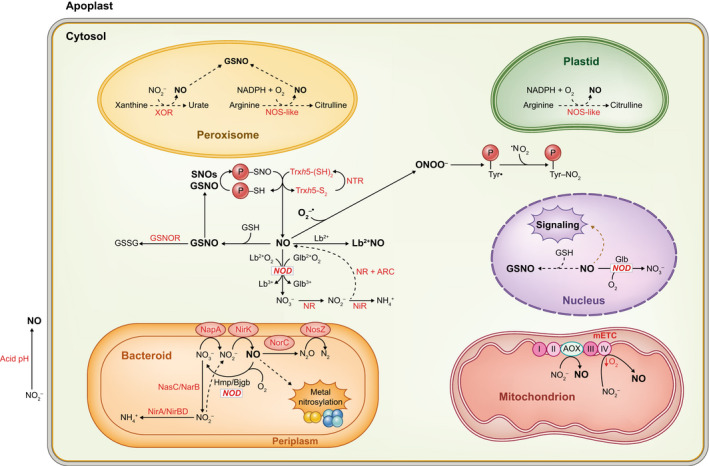
Reactive nitrogen species (RNS) metabolism in legume nodules. The scheme is based on biochemical studies and on transcriptomic and proteomic data of *Lotus japonicus* (https://lotus.au.dk; Wang *et al*., 2019, 2022) and *Medicago truncatula* (https://medicago.toulouse.inrae.fr/MtExpress; Wienkoop *et al*., 2012). Dashed lines indicate reactions that have been demonstrated only *in vitro* or that have been shown to occur in leaves. These reactions need therefore to be confirmed in nodules. Note the central role of NO, its interactions with GSH and hemoglobins, and as a precursor of peroxynitrite (ONOO^−^). Also note that the major source of NO in bacteroids is denitrification. In contrast, the assimilatory reduction of NO_3_^−^ is involved in NO production only in the free‐living rhizobia but not in *Sinorhizobium meliloti* bacteroids and needs to be confirmed in bacteroids of other rhizobial species. ARC, amidoxime reducing component; Bjgb, single‐domain hemoglobin; Glb^2+^O_2_/Glb^3+^, oxyferrous/ferric phytoglobin; GSNO, *S*‐nitrosoglutathione; Hmp, flavohemoglobin; Lb^2+^O_2_/Lb^3+^, oxyferrous/ferric leghemoglobin; mETC, mitochondrial electron transport chain; Nap, respiratory nitrate reductase; NasC/NarB, bacteroid assimilatory NR; NiR, plant nitrite reductase; NirA/NirBD, bacteroid assimilatory nitrite reductase; NirK, respiratory nitrite reductase; NOD, NO dioxygenase; NorC, respiratory NO reductase; NOS‐like, NO synthase‐like activity (arginine‐dependent); NosZ, respiratory N_2_O reductase; NR, plant nitrate reductase; NTR, NADPH‐thioredoxin reductase; SNOs, *S*‐nitrosothiols; Trx, thioredoxin; XOR, xanthine oxidoreductase.

The reductive pathways (NO_2_^−^ → NO) include NR and perhaps other molybdoproteins, the mETC, and the nonezymatic reduction in the apoplast at acid pH (Fig. 4). Genetic and pharmacological studies indicate that NR is a major source of NO in nodules (Horchani *et al*., 2011; Berger *et al*., 2020a). This is thought to occur by reduction of NO_2_^−^ to NO by the plant enzyme using NAD(P)H. In *L. japonicus* there is a single NR. The *LjNR* gene is functional in nodules in the absence of NO_3_^−^ but is upregulated following NO_3_^−^ supply (Kato *et al*., 2003). Interestingly, these authors localized *LjNR* mRNA in the infected zone and vascular bundles, which correlates with NO production, although inhibitors of NR activity were not tested (Kato *et al*., 2010). In contrast, *M. truncatula* has three functional *NR* genes. The spatiotemporal expression of *MtNR1* and *MtNR2* in nodules is correlated with NO production that occurs mainly in the nodule primordium and zone III of mature nodules; in contrast, *MtNR3* shows much lower expression than the other two genes, is specifically expressed in nodules (mainly in zones I and II of young nodules at 2 wpi and in senescent nodules at 6 wpi), and is not correlated with NO production (Berger *et al*., 2020a). The microoxic conditions of the infected cells in nodules may be conducive for NO production, which is consistent with a decrease of detectable NO in RNA interference (RNAi) lines of *MtNR*, especially of *MtNR1* (Horchani *et al*., 2011; Berger *et al*., 2020a). These authors concluded that NR activity would play a dual role, generating NO as a regulatory signal and contributing to the energy supply under the hypoxic conditions that occur inside the nodules (Berger *et al*., 2020a). In general, the reduction of NO_2_^−^ by NR may be limited to situations where NO_2_^−^ concentration is high and NO_3_^−^ and O_2_ concentrations are low because NR has much more affinity for NO_3_^−^ than for NO_2_^−^ and also because NO_2_^−^ only accumulates at relatively high levels under hypoxia (Rockel *et al*., 2002). It should be also noted that NR has multiple layers of regulation, including several PTMs, that may largely affect its activity (Lillo *et al*., 2004; Costa‐Broseta *et al*., 2021).

More recently, it has been reported that the NR of *Chlamydomonas reinhardtii* can interact with another molybdoenzyme, known as Amidoxime Reducing Component (ARC), to generate NO (Chamizo‐Ampudia *et al*., 2016). These authors showed that NR is not only able to reduce NO_3_^−^ to NO_2_^−^ but also to transfer electrons to the Mo‐cofactor of ARC, which then reduces NO_2_^−^ to NO. This reaction is operative even at millimolar concentrations of NO_3_^−^ and under normoxic conditions and led the authors to rename the ARC enzyme as NO‐forming nitrite reductase (NOFNiR) (Chamizo‐Ampudia *et al*., 2017). However, the two ARCs of Arabidopsis have been recently characterized and neither is able to generate meaningful NO at physiological NO_2_^−^ concentrations (Maiber *et al*., 2022), casting doubts about the operativity of a NOFNiR in vascular plants. Other molybdoproteins of plants like xanthine oxidoreductase, aldehyde oxidase, and sulfite oxidase can potentially reduce NO_2_^−^ to NO (Chamizo‐Ampudia *et al*., 2017, and references cited therein). However, treatment of *M. truncatula* roots with allopurinol, an inhibitor of xanthine oxidoreductase, had no effect on NO production in developing nodules (Berger *et al*., 2020a). The physiological relevance of the reactions of molybdoproteins with NO_2_^−^ merits further investigation.

Another important source of NO in plant tissues is the mETC. It is thought that NO production from NO_2_^−^ occurs at three sites: complex III (*bc1*), complex IV (cytochrome *c* oxidase), and alternative oxidase (Gupta *et al*., 2020). Under hypoxia, cytochrome *c* oxidase uses NO_2_^−^ as electron acceptor instead of O_2_ (Fig. 4). The reduction of NO_2_^−^ to NO is coupled to H^+^ translocation, keeping ATP production in the hypoxic mitochondrion. The NO diffuses to the cytosol where it is scavenged by a class 1 Glb. The resulting NO_3_^−^ is reduced to NO by cytosolic NR, so that a so‐called ‘hemoglobin‐NO cycle’ is established (Igamberdiev & Hill, 2004). Thus, the functionality of the hemoglobin‐NO cycle requires the cooperation of the cytosol and the mitochondria (Fig. 4). The ferric Glb arising from dioxygenation needs to be reduced back to the ferrous form in order to bind O_2_ and re‐enter the cycle. Several flavoproteins, including NR, as well as free flavins have been proposed as reductants of ferric to ferrous Glb (Sainz *et al*., 2013; Chamizo‐Ampudia *et al*., 2016). Notably, the NR of *C. reinhardtii* is able to provide electrons to a class 3 or truncated Glb (THB1) to maintain the hemoprotein in the functional reduced state (Chamizo‐Ampudia *et al*., 2016).

In nodules, Lbs and Glbs may potentially be involved in NO homeostasis and in the hemoglobin‐NO cycle. Because Lbs are present at millimolar concentrations, they probably play a role in scavenging NO by means of their dioxygenase activity and by directly binding NO. However, in nodules of plants experiencing severe O_2_ restrictions, as occurs in flooded soils, class 1 Glbs may have a preponderant function in the hemoglobin‐NO cycle despite being present at only micromolar concentrations because they have an extremely high O_2_ affinity. Also, the two types of hemoglobins in the ferrous state under anaerobic (or nearly) conditions display nitrite reductase activity *in vitro* by reducing NO_2_^−^ to NO. This activity is frequently overlooked and might be a source of NO in the nodule cells.

Two important RNS‐mediated PTMs are *S*‐nitrosylation and nitration. The former can be mediated by NO but the latter requires a nitrating RNS like ONOO^−^ or ^•^NO_2_ because NO itself cannot nitrate Tyr residues or a heme group. A few cases of these PTMs are known in nodules. Studies with recombinant Gpxs of the two model legumes showed that the *S*‐nitrosylation of the mitochondrial and cytosolic isoforms partially inhibits the enzyme activity (Matamoros *et al*., 2015; Castella *et al*., 2017). Thus, a crosstalk between ROS and NO at specific cellular compartments might regulate the concentration of lipid hydroperoxides and their breakdown products with potential signaling purposes. Cytosolic glutamine synthetase is inactivated by Tyr nitration and the amount of nitrated protein correlates with the decline of SNF during NO_3_^−^‐induced senescence (Melo *et al*., 2011). Lb is also susceptible to nitration in the heme and globin moiety. Heme nitration in a vinyl group was observed in soybean senescing nodules containing green Lbs (Navascués *et al*., 2012). In the globin, a Tyr residue located in the distal heme pocket is the major target of nitration (Sainz *et al*., 2015). These modifications may cause aberrant O_2_ binding and make the protein susceptible to degradation by proteases in senescing nodules.

There are also some studies about the content and/or localization of SNOs in nodules (Box 1; Table 1). Maiti *et al*. (2012) showed the presence of SNOs in peanut (*Arachis hypogaea*) nodules but, interestingly, were unable to detect NO, which is in sharp contrast with other legume nodules. If this is due to the fact that peanut is infected by bradyrhizobia through crack entry instead of through root hairs as in soybean merits further investigation. To avoid uncontrolled protein *S*‐nitrosylation while keeping NO and GSNO signaling, the concentration of GSNO needs to be fine‐tuned by GSNOR. Legumes contain two *GSNOR* genes. In *L. japonicus*, *LjGSNOR1* is predominantly expressed in leaves and roots, whereas *LjGSNOR2* is highly expressed in nodules (Matamoros *et al*., 2020). However, a specific function of LjGSNOR2 in SNF has not been demonstrated to date. The *Ljgsnor1* knockout mutants contain higher levels of SNOs in the leaves and show stunt growth, impaired nodulation, and delayed flowering and fruiting. In contrast, under optimal growth conditions, *Ljgsnor2* mutants are similar to the wild‐type, indicating that LjGSNOR1 is sufficient for normal plant development and SNF. However, a role for LjGSNOR2 under conditions of enhanced ROS and NO production, like abiotic stress and aging, cannot be ruled out.

## V. Reactive nitrogen species in bacteroids

As occurs with host nodule cells, bacteroids produce and scavenge NO. The major source of NO in bacteroids of soybean and *M. truncatula* nodules is denitrification (Sánchez *et al*., 2011; Ruiz *et al*., 2022). In *Bradyrhizobium diazoefficiens* denitrification involves the successive action of four periplasmic enzymes: nitrate reductase (NapA), nitrite reductase (NirK), nitric oxide reductase (NorC), and nitrous oxide reductase (NosZ) (Fig. 4). Some of these enzymes may be absent in other rhizobial species. For example, *R. etli* lacks Nap and Nos but has functional NirK and NorC (Gómez‐Hernández *et al*., 2011). The contribution of bacteroids to NO generation varies with the legume symbiosis. Denitrification accounts for *c*. 35% of the NO produced in *M. truncatula* nodules but reach up to *c*. 90% in soybean nodules exposed to hypoxia (Horchani *et al*., 2011; Sánchez *et al*., 2011; Ruiz *et al*., 2022). These numbers were estimated using plant and rhizobial mutants in combination with various NO detection methods. A good approach to assess NO production is to examine by EPR intact nodules formed by wild‐type and mutants deficient in denitrification enzymes. Such studies showed that soybean plants treated with NO_3_^−^ accumulated NO in nodules formed by *nirK* or *norC* mutants, especially during hypoxia caused by flooding, but not in nodules formed by the *napA* mutant of *Bradyrhizobium japonicum* (Sánchez *et al*., 2010; Calvo‐Begueria *et al*., 2018). There is now some evidence indicating that assimilatory NO_3_^−^ reduction in some bacteroids also contributes to NO production. In *R. etli* this pathway may be operative because in common bean nodules exposed to NO_3_^−^ the levels of Lb^2+^NO (marking NO production) are increased in the *norC* mutant and decreased in the *nirK* mutant (Gómez‐Hernández *et al*., 2011). In contrast, assimilatory nitrate reductase (NarB) and nitrite reductase (NirBD) of *S. meliloti* bacteroids do not contribute to NO production (Ruiz *et al*., 2022). These authors also drew the important conclusion that bacteroid NO production is not essential for symbiosis.

Another potential source of NO in bacteroids might be a NOS‐like enzyme because the genomes of some bacteria, although primarily gram‐positive, harbor a simple form of NOS (bacterial NOS or bNOS). The enzyme is a homodimer and contains an oxygenase domain like mammalian NOS but lacks the reductase domain as well as the Zn‐ and calmodulin‐binding sites (Hutfless *et al*., 2018). However, it has been recently reported that *S. meliloti* does not contain a *bNOS* gene nor expresses NOS activity (Ruiz *et al*., 2019).

Bacteroids require to keep a strict homeostasis of NO because it is not only a signal molecule required for optimal symbiotic functioning but also a potent inhibitor of nitrogenase, as shown *in vitro* (Trinchant & Rigaud, 1982) and *in vivo* experiments in which NO donors were provided to the plant (Sasakura *et al*., 2006; Kato *et al*., 2010). Thus, along with the NO sources mentioned earlier, bacteroids contain two major types of proteins that scavenge NO: the cytochrome *c* dependent‐NO reductase NorC, which reduces NO to N_2_O in the denitrification pathway, and various classes of hemoglobins. *B. japonicum* has a single domain hemoglobin (Bjgb) whereas *S. meliloti* and *M. loti* contain the flavohemoglobin Hmp. It has been reported that Bjgb plays a role in NO detoxification in bacteria grown on NO_3_^−^ and that soybean plants inoculated with a *bjgb* mutant have a less pronounced reduction of *nifH* expression and SNF when they are exposed to flooding (Salas *et al*., 2020). These authors attributed the beneficial effects of the *bjgb* mutant to a lower accumulation of NO in the nodules compared to the wild‐type nodules as a result of an induction of NorC expression and activity in *bjgb* bacteroids. The Hmp of *S. meliloti* is induced by microaerobic conditions and NO, and has been shown to be important for SNF in *M. truncatula* by using overexpressing and null mutants (Meilhoc *et al*., 2010; Cam *et al*., 2012). It was concluded that NorC (a periplasmic protein) and Hmp (a cytoplasmic protein) have no overlapping, but complementary, functions in regulating NO (Meilhoc *et al*., 2013). In addition, all these rhizobia contain a ‘truncated’ hemoglobin which is suspected to perform dioxygenation reactions and therefore modulate NO levels. However, this hypothesis has not been tested yet in nodules. An important point is that rhizobial hemoglobins require an electron donor to keep them in the functional ferrous form. Hmp can be reduced directly by NADH due to its flavin domain acting as an electron carrier, but Bjgb and truncated hemoglobins will probably require flavoproteins. Clearly, many critical questions remain open about the pathways (and their interactions) involved in NO homeostasis and signaling in the bacteroids.

## VI. Reactive sulfur species in nodule host cells

**Fig. 5:**
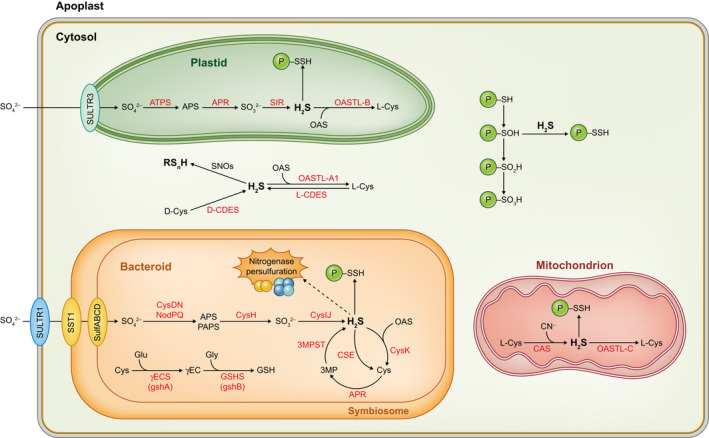
Reactive sulfur species (RSS) metabolism in legume nodules. The scheme is based on biochemical studies and on transcriptomic and proteomic data of *Lotus japonicus* (https://lotus.au.dk; Wang *et al*., 2019, 2022) and *Medicago truncatula* (https://medicago.toulouse.inrae.fr/MtExpress; Wienkoop *et al*., 2012). Some key sulfate transporters (SULTR1, SULTR3, SST1, and SulfABCD) are shown. For simplicity, oxidative reactions of thiols of protein Cys residues (P–SH) to sulfenyl (P–SOH), sulfinyl (P–SO_2_H), and sulfonyl (P–SO_3_H) groups are only indicated in the cytosol but may occur also in other cellular compartments. Similarly, the reaction of H_2_S with *S*‐nitrosothiols (SNOs) to form polysulfides (RS_*n*_H), or the reactions catalyzed by d‐CDES/l‐CDES, are shown only in the cytosol. Persulfidation of Cys residues (P–SSH) is thought to occur mainly through the reaction of P–SOH with H_2_S (Gotor *et al*., 2019). The dashed line indicates that persulfuration of nitrogenase proteins needs to be confirmed *in vivo*. 3‐MP, 3‐mercaptopyruvate; 3‐MPST, 3‐mercaptopyruvate sulfurtransferase; APR, adenosine 5′‐phosphosulfate reductase; APS, adenosine‐5′‐phosphosulfate; ATPS, ATP sulfurylase; CAS, β‐cyanoalanine synthase; CSE, cystathionine γ‐lyase; d‐CDES, d‐cysteine desulfhydrase; l‐CDES, l‐cysteine desulfhydrase; OAS, *O*‐acetylserine; OASTL‐A1, OASTL‐B and OASTL‐C, *O*‐acetylserine(thiol)lyase isoforms A1 (cytosol), B (plastids), and C (mitochondria); PAPS, 3′‐phosphoadenosine‐5′‐phosphosulfate; SIR, sulfite reductase; SNOs, *S*‐nitrosothiols.

As occurs for other plant organs, H_2_S is emerging as a key signal molecule in nodules (Fig. 5). The application of H_2_S, in the form of the typical donor sodium hydrosulfide (NaHS), to soybean plants had beneficial effects and, in particular, promoted plant growth, nodulation, and nitrogenase activity (Zou *et al*., 2019). At the molecular level, this treatment enhanced the expression of key genes for SNF such as those encoding glutamate synthase, asparagine synthase, Lb, and NifH. These authors also detected endogenous H_2_S production in young (14 dpi) and mature (28 dpi) nodules but not in nascent nodules (7 dpi) of soybean using the fluorescent probe sulfidefluor‐7 acetoxymethyl ester (SF7‐AM) and a control with the H_2_S scavenger hypotaurine (Zou *et al*., 2020; Box 1). There are other fluorescent probes commercially available for RSS. Thus, Fukudome *et al*. (2020) used HSip‐1 DA for H_2_S (Fig. 2g) and SSP4 for polysulfides (Fig. 2h). They treated *L. japonicus* plants with an RSS donor, disodium trisulfide (Na_2_S_3_), and examined the effects on roots and nodules. In roots, this treatment increased the levels of RSS and H_2_S as expected, but also decreased ROS and NO, suggesting an interplay between RSS and ROS/RNS. After 10 dpi with *M. loti*, they found an increase in the number of long ITs and in the number and weight of nodules. However, in nodules at 4 wpi, treatment of plants with Na_2_S_3_ decreased nitrogenase activity and enhanced the nodule content of poylsulfides but not of H_2_S, ROS, or NO (Fukudome *et al*., 2020). The mechanisms underlying these effects are unknown but may be multifaceted as SNF is highly sensitive to nodule manipulation and RSS can negatively affect nitrogenase or other key nodule proteins. These results reinforce the view that H_2_S and other RSS may play beneficial functions at different stages of symbiosis, but also suggest that their internal concentrations need to be tightly regulated because in excess may be detrimental to SNF.

But what are the sources of RSS in the nodule host cells? Mining in the Lotus Expression Atlas (https://lotus.au.dk; Mun *et al*., 2016) and Gene Expression Atlas of *M. truncatula* (https://medicago.toulouse.inrae.fr/MtExpress; Carrere *et al*., 2021; Dai *et al*., 2021) reveal the expression in nodules of genes encoding sulfite reductase (Lj3g3v3615680, Medtr4g077190), l‐CDES (Lj1g3v4807370, Medtr1g086070), d‐CDES (Lj0g3v0135949, Lj0g3v0216909, Medtr8g107670, Medtr3g086360), and CAS (Lj1g3v2682510, Medtr7g078070). These data are consistent with the production of H_2_S in the cytosol by l‐CDES, in the plastids by sulfite reductase and d‐CDES, and in the mitochondria by d‐CDES and CAS (Fig. 5). However, immunolocalization of these enzymes using specific antibodies and fluorescent protein tagging approaches will be necessary to unequivocally establish the sites of H_2_S synthesis in the host cells.

## VII. Reactive sulfur species in bacteroids

Both nodule host cells and bacteroids have an active sulfur metabolism, in which the biosynthesis of Cys, methionine, and GSH are of pivotal importance (Fig. 5; see review by Becana *et al*., 2018). Surprisingly, there is virtually no information on H_2_S and other RSS in bacteroids. To our knowledge, only one report has addressed this issue (Zou *et al*., 2020) but, fortunately, with fine detail. These authors generated a mutant (*ΔCSE*) of *Sinorhizobium fredii* in which the cystathionine γ‐lyase gene was deleted (Fig. 5). This is one of the two enzymes responsible for H_2_S synthesis in mammals. The *ΔCSE* mutant produced *c*. 50% of the H_2_S level found in the wild‐type strain both in the free‐living form and in soybean nodules. Upon mutant complementation, the capacity to produce H_2_S was recovered also at both stages. Notably, the *ΔCSE* mutant formed a similar number of nodules but nitrogenase activity was lower, symbiosome membranes appeared broken, and some bacteroids were deformed and contained less polyhydroxybutyrate. Furthermore, nodules and isolated bacteroids of the mutant showed increases in the contents of oxidative damage markers (carbonyls, malondialdehyde, and H_2_O_2_), the expression (mRNA and/or protein levels) of antioxidant enzymes (SOD, catalase, Prx, and Grx), and the content of thiol tripeptides. It is concluded that H_2_S acts as an antioxidant by protecting nodule cells and bacteroids from oxidative damage (Zou *et al*., 2020). It is expected that these exciting results pave the way for future achievements in the RSS field, both in generating knowledge and in agrobiotechnological applications.

## VIII. Future perspectives

The recent advances in our knowledge of the functions of ROS, RNS, and RSS in plant biology and, specifically, in the legume‐rhizobia symbiosis, prompt us to propose future paths of research. Improved methodologies to detect and quantify reactive molecules, increasing the sensitivity and specificity and avoiding artifacts, are crucial to determine the spatiotemporal profiles for generation in plant tissues of O_2_^−•^, H_2_O_2_, NO, H_2_S, and other redox signals under multiple physiological and stressful conditions. In nodules, this will clarify the contribution of organelles and bacteroids to redox homeostasis and the impact of reactive molecules on SNF. In this respect, the available information about the antioxidants and signaling pathways in the plastids, peroxisomes, and nuclei of nodule host cells is clearly insufficient, and virtually nothing is known about redox signaling in the apoplast. Many questions on NO, ONOO^−^, and SNOs still remain. To name a few, the roles of molybdoenzymes other than NR and of oxidative mechanisms in NO production; the function of bacterial hemoglobins in NO homeostasis; and the contribution of nitrosylation and nitration of proteins to metabolic regulation. How reactive molecules are perceived, transmitted, and integrated is poorly understood, and this is particularly true for RSS, which start now to be regarded as potent redox signals. Also, the transcriptional and post‐transcriptional regulation of proteins involved in the metabolism of reactive molecules must be established during nodule development and senescence. Another layer of complexity is given by the expected complex crosstalk between ROS, RNS, and RSS. Finally, discrimination between signaling and oxidative stress will improve our understanding on how plants respond to stress and will facilitate biotechnological approaches to generate plants that thrive under suboptimal growth conditions.
